# Gut Microbiota Composition and Modulation in Developmental and Epileptic Encephalopathies

**DOI:** 10.1111/ejn.70234

**Published:** 2025-08-26

**Authors:** Takwa Ammar, Fatma Abdelhedi, Leila Ammar keskes, Chahnez Charfi Triki

**Affiliations:** ^1^ Research Laboratory of Neuropediatrics LR19ES15, Child Neurology Department, Hedi Chaker University Hospital Sfax University Sfax Tunisia; ^2^ Research Laboratory of Human Molecular Genetics, Faculty of Medicine of Sfax LR99ES33 University of Sfax Sfax Tunisia; ^3^ Medical Genetics Department Hedi Chaker University Hospital Sfax Tunisia

**Keywords:** Dravet syndrome, epileptic spasms, gut dysbiosis, ketogenic diet

## Abstract

The gut microbiota (GM) is a rapidly evolving field of research that is increasingly explored in the context of various diseases. The complex interactions between the host and microbial communities play a crucial role in health and well‐being. It is now understood that the GM communicates with nearly every human organ, including the central nervous system (CNS), through the microbiome–gut–brain (MGB) axis. Furthermore, accumulating evidence suggests that pathological shifts in the GM may lead to various neurological disorders, including epilepsy. While the link between epilepsy and the MGB axis is increasingly recognized, studies investigating the impact of GM alterations in developmental and epileptic encephalopathies (DEEs) remain limited. This review highlights recent clinical and preclinical studies examining the impact of GM composition on DEEs, with a focus on infantile epileptic spasms syndrome (IESS) and Dravet syndrome (DS). Further investigation into the relationship between GM dysbiosis and the progression of DEEs is crucial for developing potential therapeutic strategies aimed at modulating the GM to alleviate seizures.

## Graphical abstract

Clinical studies link gut microbiota (GM) composition to adrenocorticotropic hormone (ACTH) treatment response. In infantile epileptic spasms syndrome (IESS) mouse models, GM modulation via antibiotics, ketogenic diet (KD), fecal transplantation, or probiotics reduced epileptic spasms, while in Dravet syndrome (DS) models, the KD showed no anti‐seizure effects despite GM dysbiosis.

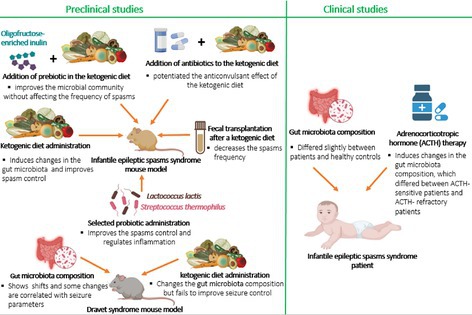

**Abbreviations**
Abx: broad‐spectrum antibiotic
ACTH: adrenocorticotropic hormone
AMPA: α‐amino‐3‐hydroxy‐5‐methyl‐4‐isooxazole‐propionic acid
ASM: anti‐seizure medication
BBB: blood–brain barrier
CD: control diet
CNS: central nervous system
CRH: corticotropin‐releasing hormone
CRHR1: CRH and its receptor type 1
CSF: cortisol in cerebrospinal fluid
DEE: developmental and epileptic encephalopathy
DS: Dravet syndrome
EE: epileptic encephalopathy
FMT: fecal microbiota transplantation
GABRE: γ‐aminobutyric acid receptor subunit epsilon
GF: germ‐free
GM: gut microbiota
HC: healthy controls
HPA: hypothalamic–pituitary–adrenal
IDO: indoleamine 2,3‐dioxygenase
IESS: infantile epileptic spasms syndrome
IL‐17a: interleukin‐17a
IL‐18: interleukin‐18
IL‐2: interleukin‐2
IL‐2R: interleukin‐2 Receptor
IL‐5: interleukin‐5
IL‐6: interleukin‐6
IL‐8: interleukin‐8
ILAE: International League Against Epilepsy
KA: kynurenic acid
KAT: kynurenine aminotransferase
KD: ketogenic diet
KEGG: Kyoto Encyclopedia of Genes and Genomes
KYN: kynurenine
LEfSe: linear discriminant analysis effect size
MGB: microbiome–gut–brain
NMDA: N‐methyl‐D‐aspartate
PRE: prebiotic
PRO: probiotic
QA: quinolinic acid
R: reference group
TDO: tryptophan 2,3‐dioxygenase
TMA: trimethylamine
TMAO: trimethylamine N‐oxide
TNF‐α: tumor necrosis factor‐α
TRP: tryptophan
WS: West syndrome
WT: wild‐type

## 1 Introduction

The core concept of epileptic encephalopathy (EE) refers to the presence of frequent epileptiform activity that directly contributes to developmental delays or regressions and may also lead to psychiatric and behavioral impairments (Scheffer et al. 2017). The 2017 revision of the International League Against Epilepsy (ILAE) classification expanded this definition to include developmental considerations (Scheffer et al. 2017). Clinically, the term developmental epileptic encephalopathy (DEE) is used when the underlying etiologies themselves contribute to developmental deficiency, while the EE process exacerbates these developmental impairments independently (Scheffer and Liao 2019; Raga et al. 2021; Zuberi et al. 2022).

DEE represents one of the most severe forms of epilepsy, typically occurring in the neonatal period and early infancy (Scheffer and Liao 2019; Raga et al. 2021; Zuberi et al. 2022). Patients with early‐onset epilepsy often exhibit increased resistance to anti‐epileptic medications and have higher mortality rates (Zuberi et al. 2022). In most cases, DEE is caused by pathogenic gene variants that lead to severe seizures, often of multiple types, accompanied by developmental disorders. Thus, even with optimal seizure control, neurocognitive abilities are expected to worsen. Despite the poor prognosis associated with DEEs, early detection and management can improve the outcomes (Scheffer and Liao 2019; Raga et al. 2021; Zuberi et al. 2022).

The gut microbiota (GM) has long been recognized for its crucial role in the bidirectional communication between the gut and the brain, known as the microbiome–gut–brain (MGB) axis (Socała et al. 2021). The GM consists of a highly diverse microbial community, comprising approximately 100 trillion microorganisms that inhabit the gastrointestinal tract. These microorganisms are classified into six major phyla: *Firmicutes*, *Bacteroidetes*, *Actinobacteria*, *Proteobacteria*, *Fusobacteria*, and *Verrucomicrobia*, with *Firmicutes* and *Bacteroidetes* being the most dominant (Hou et al. 2022). The GM, which weighs approximately 1.5 kg, encompasses around 14 families, 45 genera, and 500 distinct bacterial species (Amlerova et al. 2021).

**FIGURE 1:**
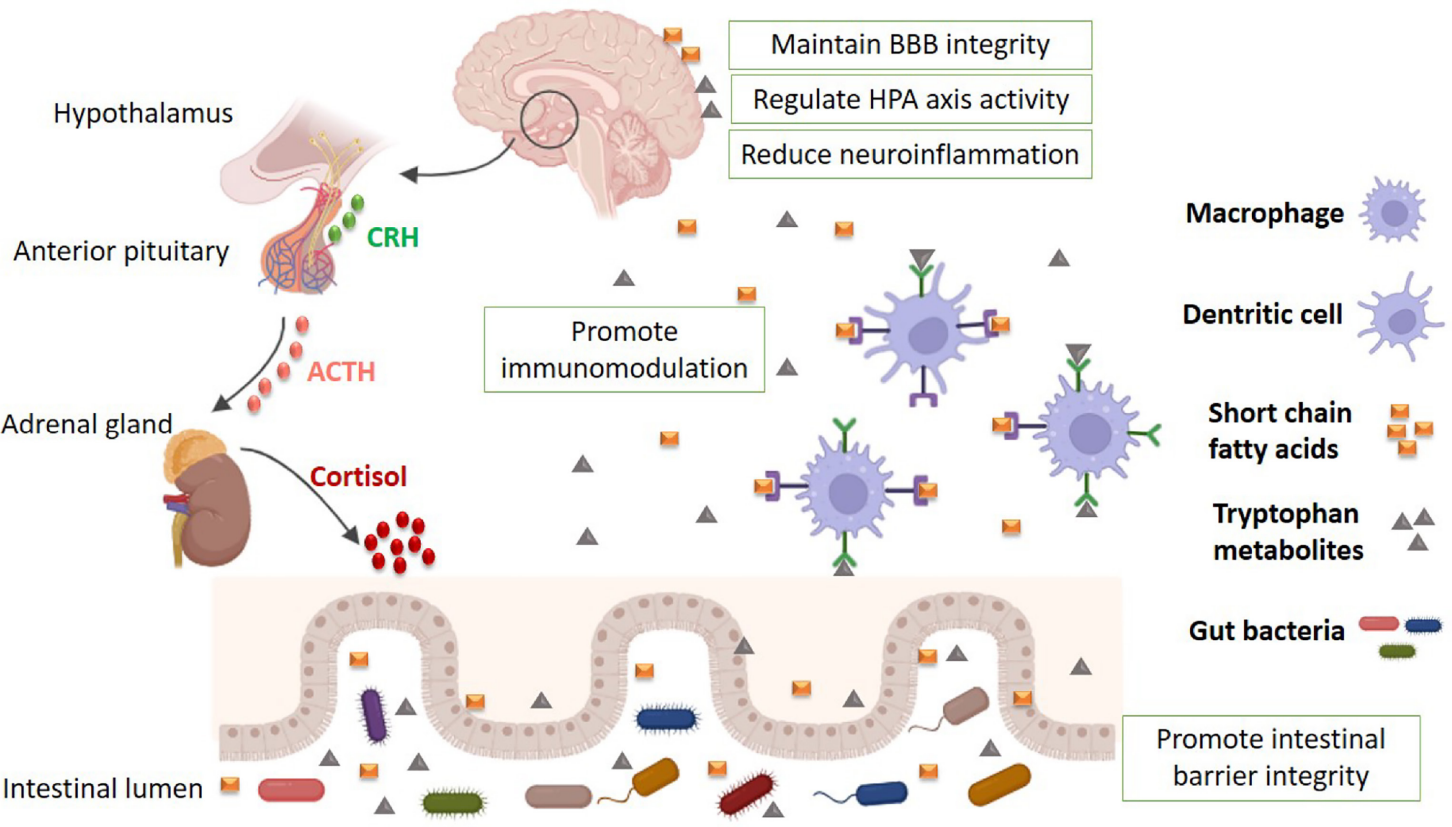
Local and distal effects of gut microbiota‐derived metabolites.

Whereas the human genome contains approximately 23,000 genes, the GM encodes more than 3 million genes, generating a vast array of metabolites that influence host physiology. The GM is considered a superorganism that interacts with the host to regulate various biological functions, including immune responses, metabolism, and neurological functions (Rinninella et al. 2019). Indeed, the GM plays a pivotal role in regulating neurodevelopment, neurotransmission, cell glial functions, cognition, and behaviors (Figure 1). Alterations in GM composition and MGB axis dysfunction have been increasingly implicated in the pathogenesis and progression of various neuropsychiatric and neurological disorders, including epilepsy (Lum et al. 2020; Socała et al. 2021) particularly in pediatric forms (Riva et al. 2024). In a recent study by Riva et al., researchers identified gut structural alterations linked to inflammation, oxidative stress, and dysbiosis in a pediatric model of acquired epilepsy, along with metabolic changes in blood and feces (Riva et al. 2024). Furthermore, the disruption of gut homeostasis and metabolic processes can subsequently impair brain function, creating a self‐reinforcing pathological cycle. Interestingly, these common alterations in epileptic models persist into adulthood, suggesting that early acute brain injury may induce long‐term GM modifications and metabolic shifts, which could contribute to epileptogenesis (Riva et al. 2024).

Although these findings are preliminary and require further validation, they underscore the role of GM composition in influencing epilepsy risk and highlight potential avenues for novel therapeutic strategies targeting GM modulation.

Since DEEs represent one of the most severe pediatric epilepsy subtypes, some authors suggest that MGB axis dysregulation may contribute to DEE progression. While the involvement of the MGB axis has been established in infantile epileptic spasms syndrome (IESS) and Dravet syndrome (DS), investigations into GM alterations in other DEE syndromes—such as CDKL5 deficiency disorder, STXBP1‐related disorders, and others—remain notably lacking.

Thus, this review provides an overview of recent clinical and preclinical studies investigating the role of GM in DEEs, with a specific focus on IESS and DS.

## 2 Infantile Epileptic Spasms Syndrome and Gut Microbiota

The term IESS was introduced in the 2022 ILAE classification to encompass both West syndrome (WS) characterized by a triad of symptoms including epileptic spasms, hypsarrhythmia, and developmental arrest or regression and other epileptic spasms that do not fully meet the diagnostic criteria of WS. IESS is classified as an early‐onset epileptic encephalopathy, with epileptic spasms typically emerging between 1 and 24 months of age (Zuberi et al. 2022; Riikonen 2023). Despite decades of extensive research, the pathogenesis of IESS remains incompletely understood.

Some patients exhibit decreased levels of adrenocorticotropic hormone (ACTH) and cortisol in cerebrospinal fluid (CSF), suggesting increased levels of corticotropin‐releasing hormone (CRH) in the central nervous system (CNS) and dysregulation of the hypothalamic–pituitary–adrenal (HPA) axis. Notably, CRH acts as a strong convulsant with high age‐specific susceptibility in early childhood. Consequently, excessive endogenous CRH release in the immature brain may contribute to IESS development (Nalin et al. 1985; Baram et al. 1995; Nagamitsu et al. 2001; Riikonen 2023).

Histological analyses of surgical specimens from IESS patients have revealed upregulated expression of CRH and its receptor type 1 (CRHR1) in epileptogenic tissue compared to controls, with a positive correlation to the frequency of epileptic spasms (Yang et al. 2017). In addition to CRH dysregulation, emerging evidence suggests that immune dysfunction plays a significant role in IESS pathogenesis. Specifically, patients exhibit elevated serum levels of pro‐inflammatory cytokines in serum, including interleukin‐2 receptor (IL‐2R), interleukin‐8 (IL‐8), and tumor necrosis factor‐α (TNF‐α), compared to healthy controls (HC) (Chen et al. 2017).

Inflammatory pathways contribute to seizure onset through a bidirectional mechanism: Seizures trigger the release of inflammatory cytokines, which may cross the blood–brain barrier (BBB), promoting neuronal hyperexcitability and exacerbating seizure severity and frequency (Riikonen 2023). The GM has emerged as a potential regulator of both the immune system and HPA axis activity (Zhang et al. 2022) (Figure 1). Furthermore, the ketogenic diet (KD), a high‐fat, low‐carbohydrate dietary intervention recommended for IESS patients who do not respond to first‐line antiepileptic drugs, is believed to exert its therapeutic effects in part by modulating GM composition (Hong et al. 2010; Olson et al. 2018).

Despite these clinical insights, the characterization of the GM profile in IESS remains a relatively new area for researchers. To date, only four studies from China have investigated the role of GM in IESS patients (Wan et al. 2021, 2024; Xu et al. 2021; You et al. 2024), while a Canadian research team has conducted three preclinical studies in rats exploring GM modulation (Mu, Choudhary, et al. 2022; Mu, Nikpoor, et al. 2022; Mu, Pochakom, et al. 2022).

### 2.1 Clinical Studies

#### 2.1.1 Alteration of Gut Microbiota Composition

The comparison of GM between IESS patients and HC revealed no significant differences in α‐and β‐diversity, suggesting that the overall microbial diversity in IESS patients does not markedly differ from that of HC (Wan et al. 2021; Xu et al. 2021; You et al. 2024) (Table 1).

**TABLE 1:** Previous clinical findings in patients with infantile epileptic spasms syndrome.

References	Cohort	Age	Methods	GM composition (IESS patients VS HC)	Prediction of microbial metabolism	GM composition after ACTH treatment	Effect of GM on ACTH treatment (ACTH‐sensitive patients VS ACTH‐resistant patients)
(Xu et al. 2021)	29 patients 29 HC	3–13 months 3–13 months	*16S rDNA* sequencing: V3–V5 hypervariable regions	No significant difference in term of α‐ and β‐diversity At phyla level: ↑*Verrucomicrobia* At genera level: ↑*Akkermansia*	↑GABRE pathway in HC compared to WS patients and after ACTH treatment	After 2 weeks: At genus level: ↓*Akkermansia*	Not determined
(Wan et al. 2021)	23 patients 21 HC	< 12 months 6–15 months	*16S rDNA* sequencing: V3–V5 hypervariable regions	No significant difference in term of α‐ and β‐diversity At genus level: ↑*Clostridium* ↓*Roseburia* ↓*Lachnospira* ↓*Lactobacillus*	↑Pathway of lipoic acid metabolism in IESS patients compared to controls	After 2 weeks: No significant difference in term of α‐ and β‐diversity At genus level: ↓*Staphylococcus*	No significant difference in term of α‐ diversity but a significant β‐diversity At genus level: ↓*Odoribacter* ↓*Phascolarctobacterium* ↓*Anaerotruncus* ↓*Mitsuokella* ↓*Robinsoniella*
(You et al. 2024)	14 patients 14 HC	1–12 months 1–12 months	*16S rDNA* sequencing: V4–V5 hypervariable regions	No significant difference in term of α‐ and β‐diversity At family level: ↓*Sutterellaceae* At genus level: ↑*Proteus* ↑*Gemmiger* ↑*Morganella* ↓*Sutterella*	Not determined	After 1 month: No significant difference in term of α‐ and β‐diversity At family level: ↓*Lactobacillaceae* At genus level: ↓*Lactobacillus*	No significant difference in term of α‐ and β‐diversity At family level: **↑** *Rikenellaceae* At genus level: **↑** *Alistipes* ↓*Megamonas* ↓*Faecalibacterium* ↓*Ruminococcus*
(Wan et al. 2024)	30 patients	Not determined	*16S rDNA* sequencing: V4–V5	Not determined	Not determined	Not determined	No significant difference in term of α‐ and β‐diversity At genus level: ↓*Clostridioides* At species level: ↓*Peptoclostridium_phage_p630P2*

Abbreviations: ACTH: adrenocorticotropic hormone; GM: gut microbiota; HC: healthy control; IESS: infantile epileptic spasms syndrome.

However, using the linear discriminant analysis effect size (LEfSe) method to identify species contributing to intergroup differences, Wan et al. (2021) reported a significantly high abundance of *Clostridium* species in the IESS group compared to the HC group (Wan et al. 2021).

This genus has previously been found to be significantly enriched in the GM of children with cerebral palsy and epilepsy, compared to HC (Huang et al. 2019). Additionally, other genera, such as *Roseburia*, *Lachnospira*, and *Lactobacillus*, were reduced in the IESS patients compared to controls (Wan et al. 2021). Importantly, *Lactobacillus* has been shown to modulate HPA axis activation in infant rats (Peng et al. 2019), suggesting that its depletion in IESS patients could contribute to disease progression (Wan et al. 2021).

Xu et al. (2021) reported a higher abundance of the *Verrucomicrobia* phylum in IESS patients compared to HC. At the genus level, only *Akkermansia*, a member of the *Verrucomicrobia* phylum, was significantly enriched in IESS patients (Xu et al. 2021) (Table 1).

Overall, the two clinical studies by Wan et al. (2021) and Xu et al. (2021) suggested that while GM composition in IESS patients differs slightly from HC, the observed alterations are primarily confined to specific bacterial genera. However, both studies included patient cohorts with heterogeneous exposure to anti‐seizure medication (ASM), a crucial factor given the growing evidence that different ASM can significantly impact GM composition and proliferation (Amlerova et al. 2021; Gong et al. 2022; Ilhan et al. 2022; Mousa et al. 2023).

Addressing this limitation, You et al. (2024) conducted a study on newly diagnosed patients who have not received any previous ASM, providing valuable insights into GM alterations independent of medication effects. Using Metastats analysis, they identified a significant reduction in the *Sutterellaceae* family in IESS patients compared to HC. The authors speculated that this decrease might be linked to disease severity. At the genus level, *Proteus*, *Gemmiger*, and *Morganella* were significantly more abundant in IESS patients, whereas *Sutterella* was markedly reduced (Table 1) (You et al. 2024).

Regarding functional prediction, Wan et al. (2021) reported a significant upregulation of lipoic acid metabolism (*lipB* and *lipA* gene expressions) in IESS patients compared to HC. The authors suggested that imbalances in GM composition and associated metabolic alterations may contribute to IESS pathogenesis (Wan et al. 2021). Notably, lipoic acid upregulation has also been linked to uncontrolled status epilepticus in a previous study by Tolunay et al. (2015) (Table 1).

Additionally, Xu et al. (2021) found that the γ‐aminobutyric acid receptor subunit epsilon (GABRE) pathway was significantly downregulated in IESS patients (Xu et al. 2021) (Table 1). However, these predictions were based on Kyoto Encyclopedia of Genes and Genomes (KEGG) pathway analysis, KEGG orthology profiling, and PICRUSt2 software. To enhance these findings, future studies should incorporate biochemical analyses of fecal and blood samples to provide a more comprehensive understanding of the metabolic changes underlying IESS.

Recently, You et al. (2024) compared HPA axis hormone levels and inflammatory cytokines between IESS patients and HC before the initiation of ASM treatment. As expected, their analysis revealed significantly elevated levels of CRH and pro‐inflammatory cytokines including interleukin‐2 (IL‐2), interleukin‐6 (IL‐6), and interleukin‐17a (IL‐17a) in the serum of IESS patients compared to the control group. These findings are consistent with previous studies, further supporting the role of HPA axis dysregulation and systemic inflammation as key biochemical mechanisms underlying the pathogenesis of IESS (Türe et al. 2016; Riikonen 2023).

Despite the valuable insights provided by these clinical studies, certain limitations must be acknowledged. First, the sample size was relatively small, which may limit the generalizability of the findings. Second, the use of *16S rDNA* gene sequencing for GM analysis restricts taxonomic resolution to the genus level and above. Thus, future research should aim to include larger patient cohorts and employ metagenomic sequencing technologies to enhance the accuracy and depth of GM characterization.

Additionally, accumulating evidence suggests that antibiotic use can significantly alter GM composition. A study by Palleja et al. (2018) on healthy adults reported that GM recovery following antibiotic exposure takes approximately 6 weeks, with some species remaining undetectable even after 180 days of treatment (Palleja et al. 2018). However, in their clinical study, You et al. (2024) ensured that recruited patients had not taken antibiotics in the 2 weeks prior to study enrollment (You et al. 2024). Given the potential impact of antibiotics on GM, future studies should consider investigating newly diagnosed patients who have not received antibiotic treatment to minimize confounding factors.

#### 2.1.2 Effect of Adrenocorticotropic Hormone Therapy on Gut Microbiota

ACTH hormonotherapy is one of the most effective treatments for managing IESS. Although its antiepileptic effects have been attributed to several biological processes, the precise mechanism of action remains unclear (Wan et al. 2021). ACTH is well recognized for its immunomodulatory properties, primarily mediated through stimulation of adrenal glucocorticoid production. Notably, treatment with prednisone (a synthetic glucocorticoid) in IESS patients has been shown to significantly reduce elevated pro‐inflammatory cytokines in the serum, decrease the frequency of ictal clusters, and improve both EEG patterns and the developmental quotient (Chen et al. 2017).

While ACTH exerts its antiepileptic effects by targeting immune dysfunction, its mechanism of action is not entirely anti‐inflammatory (Shandra et al. 2017). Previous studies have speculated that ACTH mitigates epileptic spasms by downregulating the release of the proconvulsant factor CRH in the CNS through negative feedback mechanisms (Baram et al. 1995; Nagamitsu et al. 2001; Riikonen 2023). Moreover, given the bidirectional communication between the GM and the HPA axis, some researchers have suggested that the MGB axis may play a role in ACTH anticonvulsant effects (Farzi et al. 2018; Xu et al. 2021) (Figure 1).

Following 14 days of ACTH treatment, assessments of α‐ and β‐diversity revealed no significant differences in GM composition (Wan et al. 2021; You et al. 2024). Nevertheless, the abundance of *Staphylococcus* (Wan et al. 2021) and *Akkermansia* (Xu et al. 2021) decreased significantly in patient cohorts following ACTH hormonotherapy. The potential involvement of the *Akkermansia* genus in epilepsy was previously discussed by Peng et al., who reported increased levels of this genus in drug‐resistant epilepsy patients compared to drug‐sensitive individuals and healthy controls (Peng et al. 2018).

Moreover, in the study by You et al., the abundance of *Lactobacillus* and *Lactobacillaceae* decreased as the duration of ACTH treatment increased, with significantly lower levels observed after 1 month of treatment compared to both the 2‐week treatment group and the HC group (Table 1). Based on these findings, the authors hypothesized that as symptoms improved with treatment, the GM composition of children with IESS shifted toward a predominance of beneficial bacteria (You et al. 2024). These clinical studies suggest that after short‐term ACTH treatment (2 weeks or 1 month), only specific bacterial genera showed significant changes in abundance.

Therefore, further research with larger patient cohorts and longer treatment durations is needed to better understand the effects of ACTH on GM composition.

On the other hand, the interaction between ASMs and their metabolites with the composition of the GM has prompted several studies to investigate the role of gut bacterial communities in determining ASMs response. For instance, Riva et al. recently demonstrated that drug‐resistant epilepsy patients exhibit reduced abundance of butyrate‐producing bacteria—particularly the *Eubacterium siraeum* group—compared to drug‐sensitive patients (Riva et al. 2025).

Similarly, in studies of ACTH hormone therapy, researchers have classified patients into two distinct groups: ACTH‐sensitive group and ACTH‐resistant group (Wan et al. 2021, 2024; You et al. 2024) (Table 1). The comparison between these two groups has yielded variable findings. Analysis of the bacterial communities revealed no significant differences in α‐diversity between ACTH‐sensitive and ACTH‐resistant groups across studies (Wan et al. 2021, 2024; You et al. 2024). However, in terms of β‐diversity, Wan et al. found a significant difference between these two groups (Wan et al. 2021). In this study, the ACTH‐sensitive group (*n* = 18) exhibited a decrease in the populations of *Odoribacter*, *Phascolarctobacterium*, *Anaerotruncus*, *Mitsuokella*, and *Robinsoniella* compared to the ACTH‐resistant group (*n* = 5) (Wan et al. 2021). Importantly, the genus *Anaerotruncus* showed a significant upregulation accompanied by excessive activation of the HPA axis in the stressed prenatal mice (Golubeva et al. 2015). Additionally, Wan et al. reported that *Bifidobacterium* abundance was higher in the ACTH‐sensitive group than in the ACTH‐resistant group (Wan et al. 2021). This aligns with previous findings showing that recolonization of germ‐free (GF) mice with *Bifidobacterium infantis* normalizes excessive HPA axis activation (Sudo et al. 2004).

In a recent study by You et al., Metastats analysis showed that in the ACTH‐sensitive group (*n* = 9) *Alistipes* and *Rikenellaceae* abundance were significantly enriched, whereas *Megamonas*, *Faecalibacterium*, and *Ruminococcus* abundance were significantly reduced compared to the ACTH‐resistant group (*n* = 5) (You et al. 2024). Similarly, increased *Ruminococcus* abundance has also been observed in drug‐refractory epilepsy patients (Peng et al. 2018). Another study using Metastats and LEfSe analysis reported that the abundance of *Clostridioides* genus and *Peptoclostridium_phage_p630P2* (a species within *Clostridioides*) were significantly more abundant in the ACTH‐resistant group (*n* = 16) compared to the ACTH‐sensitive group (*n* = 14) before treatment (Wan et al. 2024). Based on these findings, the authors hypothesized that the elevated levels of *Clostridioides* in IESS patients may counteract the anticonvulsant effects of ACTH. These observations suggest that restoring GM balance could be a potential adjuvant therapy for managing epileptic spasms in infants (Wan et al. 2021).

The discrepancies observed across studies may be attributed to the heterogeneity of patients, particularly in terms of ASM used alongside ACTH treatment. Additionally, variations in the criteria used to evaluate treatment response could have contributed to the inconsistencies in findings. Future research should aim to standardize patient selection criteria and incorporate larger, well‐defined cohorts to improve the reliability of GM‐related analyses in IESS.

### 2.2 Preclinical Studies: Modulation of Gut Microbiota

#### 2.2.1 Effects of Ketogenic Diet

The KD, characterized by high fat and low carbohydrate intake, has been shown to mitigate seizures in drug‐resistant epilepsy patients (Attaye et al. 2021). In two recent studies, Mu et al. reported that the KD increased α‐diversity and enriched the GM community in intractable IESS animal models compared to those fed a control diet (CD) (Mu, Choudhary, et al. 2022; Mu, Nikpoor, et al. 2022) (Table 2). However, findings from other studies involving children and adult mice with epilepsy have been inconsistent, with some reporting a decrease (Paoli et al. 2019) or no significant change in α‐diversity following KD (Peng et al. 2018).

**TABLE 2:** Summary of preclinical results obtained by the study of infantile epileptic spasms syndrome animal model.

References	Objectives	Effects on GM diversity	Effects on GM communities	Effects on metabolic	Predicted metagenomic function
(Mu, Choudhary, et al. 2022)	Effect of KD in IESS model	**↑**α‐Diversity in KD group compared to CD group	At species level: **↑** *Streptococcus infantis* **↑** *Streptococcus lactarius* **↑** *Streptococcus thermophilus* **↑** *Lactococcus lactis* **↓** *Lactobacillus johnsonii* **↓** *Escherichia coli* in KD group compared to CD group	In the serum: ↑Level of TRP, pyridoxal 5′‐phosphate, KA, phenylalanine, and tyrosine ↓Level of 5‐hydroxytryptophan and glutamylaspartic acid in KD group compared to CD group	**↑**Gene abundance of TRP, tyrosine, and phenylalanine metabolism in KD group compared to CD group
(Mu, Nikpoor, et al. 2022)	Effect of KD in IESS model	**↑**α‐Diversity in KD compared with CD group	At phyla level: **↓** *Actinobacteria* At species level: **↑** *Streptococcus thermophilus* **↑** *Lactococcus lactis* **↓** *Ligilactobacillus animalis* in KD group compared to CD group	In the serum the and hippocampus: **↑**The level of β‐hydroxybutyric acid, lipoyl‐GMP, glycine, and N‐acetyldopamine, In the serum: **↑**Taurocholic acid **↑**Glutarylcarnitine **↑**Acetoacetic acid In the hippocampus: **↑**3‐Hydroxybutyrylcarnitine **↑**Inoleylcarnitine, **↑**Glutathione in KD group compared to CD group	**↑**Arachidonic acid **↑**Ascorbate **↑**Thiamine and **↑**Pantothenate metabolism in KD group compared to CD group
(Mu, Nikpoor, et al. 2022)	Effect of antibiotic addition to KD	**↑**α‐Diversity in (KD + Abx) group compared to CD group	At genus level: **↑** *Streptococcus* **↑** *Lactococcus* **↓** *Enterococcus* in (KD + Abx) group compared to CD group	Not determined	Not determined
(Mu, Choudhary, et al. 2022)	Effect of antibiotic addition to KD	Not determined	At genus level: **↑** *Lactococcus* **↑** *Streptococcus* **↓** *Enterococcus* **↓** *Lactobacillus* in (KD + Abx) group compared to KD group	In the serum: **↑**Level of KA **↑**Level of xanthurenic acid **↑**KA/KYN ratio and KAT activity in (KD + Abx) group compared to CD group **↑**Level of indole‐3‐acetamide in (KD + Abx) group compared to KD group	**↑**TRP 2,3‐dioxygenase **↑**TRP 2‐monooxygenase in (KD + Abx) group compared to KD group
(Mu, Nikpoor, et al. 2022)	Effect of probiotic (*S thermophilus* and *L lactis mixture*)	Not determined	At species level: **↑** *Lactococcus lactis* in (CD + PRO) group compared to CD group	In the hippocampus: **↓**Serine **↓**Ornithine **↑**Glutathione **↓**Allantoin **↓**Hypoxanthine **↑**Inosine 2′‐phosphate **↑**Adenosine monophosphate in (CD + PRO) group compared to CD group	**↑**Metabolism of arachidonic acid in (CD + PRO) group compared to CD group
(Mu, Pochakom, et al. 2022)	Effect of prebiotic (PRE, oligofructose‐enriched inulin) addition to KD	**↑**α‐Diversity in (KD + PRE) group compared to R group	At phyla level: **↓** *Firmicutes* **↑** *Proteobacteria* in (KD + PRE) group compared to KD group and R group At species level: ↑ *Lactobacillus johnsonii* in (KD + PRE) compared to KD group Appareance of *Bifidobacterium pesudolongum*, which was not present neither in R nor in KD animals	In the serum: **↓**Ketone **↑**Glucose in (KD + PRE) group compared to KD group In the hippocampus: Relative to KD, the KD + PRE increased the concentrations of glutathione, CMP‐N‐acetyl‐β‐neuraminic acid, and adenosine monophosphate	Not determined

Abbreviations: Abx: broad‐spectrum antibiotic; CD: control diet; GM: gut microbiota; IESS: infantile epileptic spasms syndrome; KD: ketogenic diet; PRE: prebiotic; PRO: probiotic; R: reference group; TRP: tryptophan.

This discrepancy may be attributed to limitations in the artificial IESS model, particularly as lesion‐induced spasms occur in neonatal mice before GM maturation (Mu, Choudhary, et al. 2022).

At the phylum level, the KD significantly reduced the relative abundance of *Actinobacteria* (*p* = 0.029) compared to the CD group (Mu, Nikpoor, et al. 2022), a finding consistent with previous research by Qy et al., which demonstrated that lower carbohydrate intake during KD is associated with reduced *Actinobacteria* abundance (Qy et al. 2020).

The most notable microbial changes following KD included a significant increase in the abundance of *Streptococcus thermophilus* (*p* = 0.022) and *Lactococcus lactis* (*p* = 0.044), along with a decrease in *Ligilactobacillus animalis* (*p* = 0.017) compared to the CD group (Mu, Nikpoor, et al. 2022) (Table 2).

Similarly, another study by Mu, Choudhary, et al. (2022) noted that at the species level, the KD upregulated the abundances of *Streptococcus infantis* (*p* = 0.001), *Streptococcus lactarius* (*p* < 0.001), *Streptococcus thermophilus* (*p* < 0.001), and *Lactococcus lactis* (*p* = 0.005), while decreasing the abundances of *Lactobacillus johnsonii* (*p* = 0.019) and *Escherichia coli* (*p* = 0.011) in IESS rats fed the KD compared to those on the CD (Mu, Choudhary, et al. 2022) (Table 2).

Importantly, both *Lactococcus lactis* and *Streptococcus thermophilus* play key roles in tryptophan (TRP) metabolism by producing aromatic amino acid transaminase, which metabolizes TRP into indole‐3‐lactate. Additionally, *Lactococcus lactis* species can produce tryptophan 2,3‐dioxygenase (TDO), an enzyme that metabolizes TRP into kynurenine (KYN). Functional predictions using PICRUSt analysis further supported these metabolic changes, showing an increase in microbial genes involved in TRP, tyrosine, and phenylalanine metabolism in IESS models fed the KD compared to the CD group (*p* = 0.039) (Mu, Choudhary, et al. 2022).

Moreover, PICRUSt‐based metagenomic predictions suggested that the KD enhances antioxidant status by upregulating ascorbate and thiamine metabolism (*p* < 0.05) (Mu, Nikpoor, et al. 2022). In line with this prediction, metabolic analysis findings indicated that the KD upregulated serum levels of glycine and N‐acetyldopamine (*p* < 0.05), both of which are known for their antioxidant and anti‐inflammatory properties in the serum and hippocampus. These metabolic shifts led researchers to hypothesize that the antioxidant effects induced by the KD contribute to reduced seizure frequency (Mu, Nikpoor, et al. 2022).

In a study by Mu, Choudhary, et al. (2022), the KD significantly increased serum levels of TRP, pyridoxal 5′‐phosphate, kynurenic acid (KA), phenylalanine, and tyrosine, while decreasing 5‐hydroxytryptophan and glutamylaspartic acid in IESS animals compared to the CD group. These findings suggest that the TRP‐KYN metabolic pathway may play a role in the anticonvulsant mechanism of the KD (Mu, Choudhary, et al. 2022).

Dysregulation of this pathway has been involved in various neurological disorders due to its dual role in neuroprotection or neurotoxicity.

**FIGURE 2:**
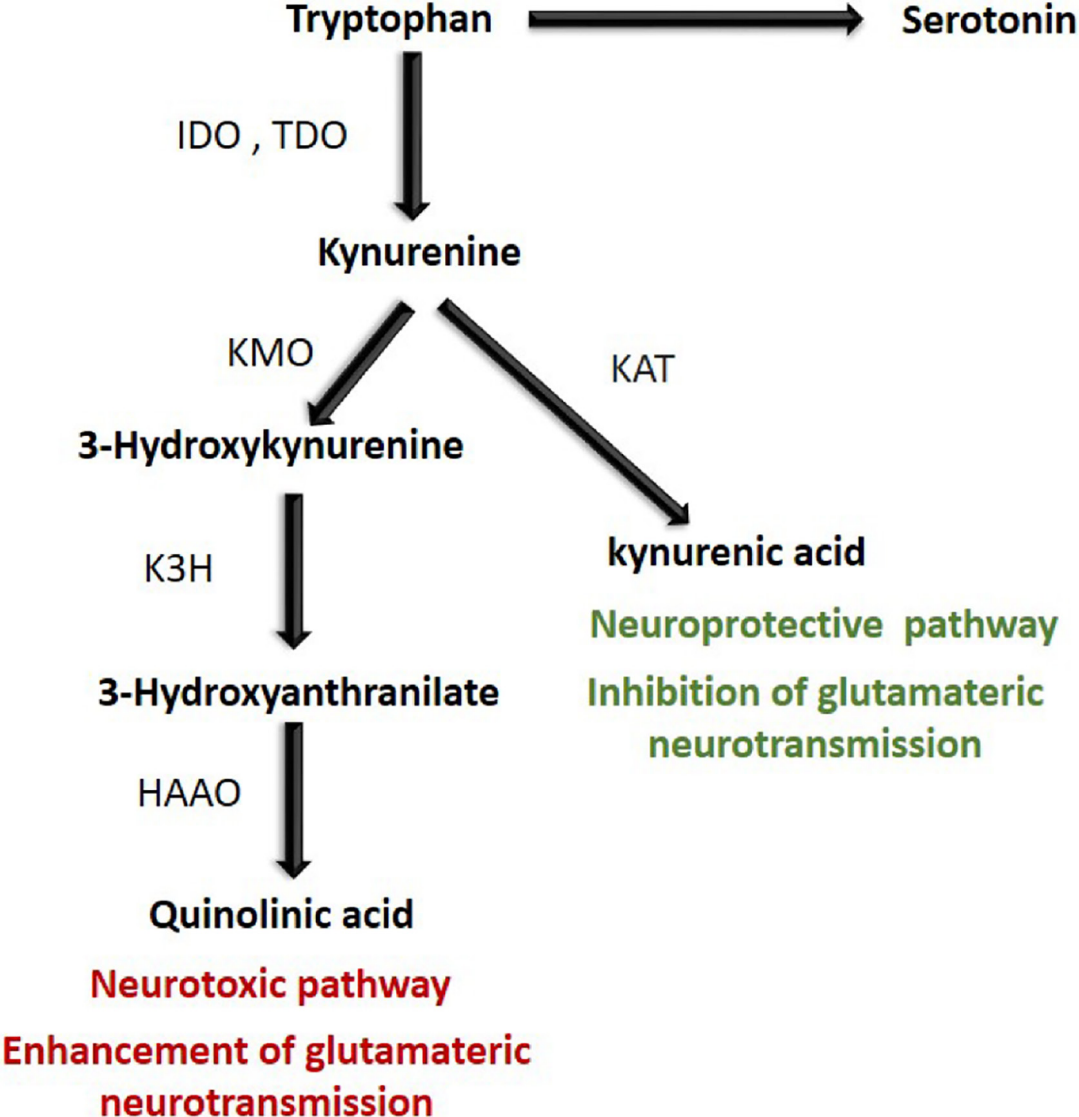
Tryptophan kynurenine pathway. HAAO: 3‐hydroxyanthranilate 3,4‐dioxygenase; IDO: indoleamine 2,3‐dioxygenase; K3H: kynurenine 3‐hydroxylase; KAT: kynurenine aminotransferase; KMO: kynurenine 3‐monooxygenase; TDO: tryptophan 2,3‐dioxygenase.

The TRP‐KYN pathway, responsible for approximately 95% of TRP catabolism, is regulated by two key enzymes: TDO and indoleamine 2,3‐dioxygenase (IDO), both of which catalyze TRP conversion into KYN. While IDO plays a more dominant role in TRP metabolism than TDO, the pathway can proceed via two distinct routes (Figure 2). The first leads to the production of KA, a neuroprotective metabolite generated by kynurenine aminotransferase (KAT). As an endogenous glutamate receptor antagonist, KA inhibits neurotransmission via N‐methyl‐D‐aspartate (NMDA), kainate, and α‐amino‐3‐hydroxy‐5‐methyl‐4‐isooxazole‐propionic acid (AMPA) receptors. The second pathway, which is more active when IDO is upregulated, produces neurotoxic metabolites such as quinolinic acid (QA), which enhances glutamatergic neurotransmission and contributes to neuronal dysfunction (Mazarei and Leavitt 2015; Ye et al. 2019) (Figure 2).

Moreover, the KD group showed a higher KA/KYN ratio (*p* = 0.036) than CD rats, indicating increased KA production (Mu, Choudhary, et al. 2022).

Supporting the anticonvulsant role of KA, previous studies have reported elevated KA levels following KD in patients with refractory epilepsy (Żarnowska et al. 2019), as well as increased KA formation in rat cortical slices treated with antiseizure medications (Kocki et al. 2006). Moreover, a recent study found that IESS patients have reduced KA levels and a lower KA/KYN ratio in CSF (Yan et al. 2022). In contrast, the KD group showed a decreased KYN/TRP ratio (*p* = 0.018), suggesting reduced IDO1 enzyme activity.

To further investigate the role of IDO1 downregulation in seizure control, researchers tested two antagonists (1‐methyltryptophan and minocycline) to inhibit the production of KYN from TRP by IDO1. As expected, compared to IESS rats fed the CD, the group treated with KD in combination with either 1‐methyltryptophan (1‐MT) or minocycline showed a significant reduction in spasm frequency (*p* < 0.001) and increased KA levels in both serum and the hippocampus. Interestingly, seizure reduction was also observed in the CD group treated with IDO1 antagonists. Overall, these findings indicate that the anticonvulsant effect of the KD may be mediated, at least in part, by modulating TRP metabolism toward increased KA production (Mu, Choudhary, et al. 2022). Further research is needed to elucidate the precise mechanisms underlying these metabolic shifts and their potential therapeutic implications for epilepsy management.

#### 2.2.2 Effects of Fecal Microbiota Transplantation

In the study by Mu, Choudhary, et al. (2022), fecal microbiota transplantation (FMT) from a KD group to a CD group in IESS animal models resulted in a reduction of spasms frequency, bringing its levels comparable to those observed in the KD group. This reduction was also associated with a decrease in the KYN/TRP ratio compared to the non‐transplanted CD group, suggesting that FMT following KD may help reduce the occurrence of spasms, potentially through shifts in TRP metabolism.

However, since the GM composition was not analyzed after FMT, it remains unclear whether the observed effects were due to microbiome shifts or the presence of residual dietary metabolites from the KD (Mu, Choudhary, et al. 2022).

#### 2.2.3 Effects of Antibiotics Added to the Ketogenic Diet

To investigate the relationship between the occurrence of epileptic spasms and the GM composition, a broad‐spectrum antibiotic (Abx) was administered to IESS models in combination with the KD. This treatment further improved the efficacy of the KD in reducing spasm frequency (*p* = 0.039 for KD + Abx vs. KD). Notably, mice receiving both KD and Abx exhibited the highest α‐diversity compared to the CD group (Mu, Nikpoor, et al. 2022). While no significant changes were observed at the phylum level, the combined treatment led to an upregulation in the relative abundances of certain beneficial genera, primarily *Streptococcus* (*p* = 0.002) and *Lactococcus* (*p* < 0.001), while downregulating *Lactobacillus* (*p* = 0.077) and *Enterococcus* (*p* = 0.015) compared to the CD group (Table 2).

Overall, the researchers proposed that the combined effects of KD and Abx reduced spasms frequency either by eliminating harmful bacterial species that may exacerbate seizures or by promoting the growth of beneficial species that contribute to seizure reduction (Mu, Nikpoor, et al. 2022).

In another study, Mu, Choudhary, et al. (2022) similarly reported that compared to the KD alone, the addition of antibiotics to the KD increased the relative abundances of the same discriminant genera, *Lactococcus* (*p* = 0.001) and *Streptococcus* (*p* = 0.001), while significantly decreasing the abundances of *Enterococcus* (*p* < 0.001) and *Lactobacillus* (*p* = 0.016) (Mu, Choudhary, et al. 2022) (Table 2).

In addition, the researchers found that the combined treatment appeared to enhance the enzymatic activity of KAT, which catalyzes the conversion of KYN to the neuroprotective metabolite KA (Figure 2). This was indicated by a significant increase in the KA/KYN ratio (*p* = 0.009) and in the relative level of KA (*p* < 0.001) in the serum of rats treated with KD and antibiotics compared to those fed the CD diet (Table 2). Based on these findings, the authors postulated that the addition of antibiotics potentiated the anticonvulsant effect of the KD by modulating the TRP‐KYN pathway metabolism, leading to reduced KYN levels and enhanced KA production (Mu, Choudhary, et al. 2022).

#### 2.2.4 Effects of Probiotic Administration

In a pivotal study by Mu, Nikpoor, et al. (2022), the authors hypothesized that manipulating the GM to mimic the therapeutic effects of the KD could improve seizure control in rodent models of IESS (Mu, Nikpoor, et al. 2022).

To test this hypothesis, they administered a targeted probiotic (PRO) combination consisting of *Streptococcus thermophilus* and *Lactococcus lactis* , two species that had previously been shown to increase following KD and antibiotic treatments and are known for their psychobiotic and anti‐inflammatory properties. Their findings demonstrated that supplementation with this PRO mixture in rodents fed a CD led to several beneficial effects. Specifically, PRO administration reduced spasms recurrence, decreased serum levels of proinflammatory cytokines, including IL‐6, Interleukin‐18 (IL‐18), and TNF‐α, and upregulated the anti‐inflammatory cytokine Interleukin‐5 (IL‐5), along with antioxidant metabolites involved in glutathione metabolism. Additionally, significant anti‐inflammatory and antioxidant effects were observed in hippocampal tissues following PRO treatment (Table 2). Based on these results, the authors proposed that the selected PRO formulation may regulate chronic inflammation and oxidative stress, thereby exerting an anticonvulsant effect (Mu, Nikpoor, et al. 2022).

Metagenomic analysis further revealed that PRO supplementation increased the abundance of *Lactococcus lactis* compared to the CD group. Functional predictions suggested that PRO administration, whether combined with KD or CD, upregulated microbial pathways involved in arachidonic acid metabolism (*p* < 0.05) compared to the CD group. Overall, these findings suggest that targeted PRO administration may serve as a complementary therapeutic approach in IESS by modulating the GM and host metabolism to enhance seizure control (Mu, Nikpoor, et al. 2022).

#### 2.2.5 Effects of Prebiotic Inclusion in the Ketogenic Diet

In a recent study, Mu, Pochakom, et al. (2022) investigated the effects of adding a prebiotic (PRE) fiber, specifically oligofructose‐enriched inulin, to the KD on spasms mitigation. Researchers evaluated spasms frequency, GM composition, and metabolic profiles in both serum and hippocampus to evaluate the impact of PRE inclusion after KD initiation (Mu, Pochakom, et al. 2022).

Following spasms induction, IESS models were randomly assigned to either the KD group or the KD + PRE group. These groups were compared to a reference group (R) consisting of breastfed animals without IESS.

The assessment of seizure frequency revealed no significant difference between the KD and KD + PRE groups (*p* = 0.665). However, PRE supplementation led to a significant increase in blood glucose levels and a decrease in ketone levels (*p* < 0.001), which is known to play a major role in the antiseizure effects of the KD. Given that the reduction in ketones following PRE addition did not diminish the antiseizure effects of KD, the authors speculate that the KD does not rely solely on circulating ketones to control spasms. Despite the lack of impact on seizure frequency, GM characterization showed that the combination of PRE and KD significantly enhanced bacterial richness compared to the R group (Mu, Pochakom, et al. 2022). Specifically, the KD + PRE group exhibited a decreased relative abundance of *Firmicutes* and an increased abundance of *Proteobacteria* (*p* < 0.05) compared to both the KD and R groups.

Additionally, in comparison to the KD group, the KD + PRE group showed a reduction in *Ligilactobacillus animalis* (*p* = 0.124) and *Lactococcus lactis* (*p* = 0.085), while *Lactobacillus johnsonii* was significantly increased (*p* = 0.020).

Interestingly, the addition of PRE promoted the growth of *Bifidobacterium pseudolongum*, a species that was absent in both the R and KD groups (Mu, Pochakom, et al. 2022). Since PRE serves as a potential source of energy for the GM, its inclusion likely induces the growth of beneficial bacteria, mainly those within *Bifidobacterium* and *Lactobacillus* (Mayengbam et al. 2019). Based on these findings, the authors concluded that while PRE supplementation enhances microbial community, it does not impact spasms frequency (Mu, Pochakom, et al. 2022).

## 3 Dravet Syndrome and Gut Microbiota

DS, previously known as Severe Myoclonic Epilepsy of Infancy, is a rare early‐onset EE. Affected individuals typically exhibit normal development until seizure onset, which usually occurs between 4 and 8 months of age.

Seizures are often triggered by febrile episodes, and patients experience prolonged focal clonic or generalized clonic seizures (Wheless et al. 2020). During the subsequent worsening phase, which occurs between one and four years old, patients develop additional seizure types, primarily myoclonic and atypical absence seizures (Wheless et al. 2020; Zuberi et al. 2022). Over time, seizures typically become pharmacoresistant, with cognitive and behavioral impairments often arising by the second year of life. Non‐seizure‐related symptoms, such as ataxia and crouching gait problems, may also develop. Importantly, in over 80% of cases, DS is caused by *de novo* mutations in the *SCN1A* gene, which encodes the α‐subunit of voltage‐gated sodium channels expressed in most neurons of the CNS (Zuberi et al. 2022).

Despite the well‐characterized genetic basis of DS, little research has explored the role of the GM in this disorder. To date, only one preclinical study, conducted by Miljanovic and Potschka, has investigated GM composition in a DS mouse model with *SCN1A* deficiency compared to wild‐type (WT) controls. The study also examined the effects of dietary interventions, specifically a KD versus a CD, on GM composition. Fecal samples were collected at two different time points: first, at baseline before dietary treatment and second, after 6 weeks of dietary intervention, 1 day prior to sacrifice. GM composition was analyzed using bacterial *16S rDNA* gene (V3‐V4) sequencing (Miljanovic and Potschka 2021).

### 3.1 Gut Microbiota Composition

The analysis of α‐diversity showed a reduction in the DS model compared to WT before any dietary intervention, suggesting that DS may influence GM composition. Baseline microbiome analysis further indicated that, compared to WT, DS models exhibited a lower abundance of *Bacteroidetes* and a higher abundance of *Firmicutes*. Notably, an increased *Firmicutes‐to‐Bacteroidetes* ratio, a hallmark of gut dysbiosis, was observed in DS animals (Miljanovic and Potschka 2021).

This finding aligns with a study by Hoffman et al., which reported that an elevated *Firmicutes*‐to‐*Bacteroidetes* ratio could lead to BBB dysfunction and neuroinflammation in older models. Such detrimental CNS changes are linked to cognitive impairment and heightened anxiety (Hoffman et al. 2017).

Furthermore, an increased abundance of *Firmicutes* may stimulate the production of trimethylamine (TMA) and its metabolite trimethylamine N‐oxide (TMAO). Notably, TMAO, derived from GM, can cross the BBB, activate glial cells, induce neuroinflammation, and contribute to cognitive decline in mouse models (Brunt et al. 2020). Thus, the authors hypothesized that GM alterations associated with DS may exacerbate cognitive disorders in affected individuals (Miljanovic and Potschka 2021).

Additionally, a trend toward upregulation of *Deferribacteres* was observed in DS mice. Interestingly, this phylum was strongly correlated with increased seizure frequency and duration in DS models.

At the taxonomic level, DS mice exhibited a decreased abundance of the *Barnesiella* genus, along with a tendency for increased abundance of the *Lachnoclostridium* and *Mucispirillum* genera, both of which were positively correlated with seizure parameters. Referring to other studies, the authors mentioned that upregulation of *Deferribacteres* and its genus *Mucispirillum* has been previously associated with pro‐inflammatory responses involving IL‐6. Given that excessive inflammation is a key driver of epileptogenesis and the worsening of cognitive, behavioral, and neurological comorbidities in DS, these microbiome alterations may play a role in disease progression (Miljanovic and Potschka 2021).

### 3.2 Effects of Ketogenic Diet on Gut Microbiota

After exposure to the KD, species diversity analysis revealed an increase in both WT and DS mice compared to those on the CD.

In terms of phyla, KD led to an upregulation of *Firmicutes* and a downregulation of *Bacteroidetes*, resulting in an increased *Firmicutes*‐to‐*Bacteroidetes* ratio (Miljanovic and Potschka 2021).

KD also altered GM composition by increasing the abundance of *Acetatifactor*, *Alloprevotella*, and *Enterorhabdus* genera, while decreasing the abundance of *Barnesiella*, *Lactobacillus*, and *Bacteroides* genera in both WT and DS models. Additionally, *Lachnoclostridium* and *Eisenbergiella* genera showed a trend toward increased abundance.

Notably, the abundance of *Lachnoclostridium* genus was negatively correlated with seizure frequency (R = −0.65) and seizure duration (R = −0.55). Meanwhile, shifts in *Lactobacillus*, *Bacteroides*, and *Enterorhabdus* abundance were correlated with hippocampal levels of ketosis‐related β*‐hydroxybutyrate* in DS mice (R = 0.58, −0.58, and 0.64, respectively) (Miljanovic and Potschka 2021).

Despite these significant microbial changes, KD did not lead to improvements in seizure parameters or behavioral impairments in DS animals. However, gait disturbances showed improvement, though this effect was not directly correlated with the microbiome shifts induced by KD. The lack of seizure control following KD contrasts with previous findings, prompting the authors to identify potential influencing factors, including KD duration, dietary composition, and methodologies used for GM analysis.

Overall, this study was the first to characterize gut dysbiosis in DS mice and document the GM changes following KD. While these findings remain preliminary, they nevertheless emphasize the concept that even the monogenic form of DEE exhibits GM alterations. In addition, the inability of KD to reduce seizures underlines both the complexity of MGB interactions and the need for deeper investigation to clarify the mechanisms underlying the effects of KD on DS (Miljanovic and Potschka 2021).

## 4 Conclusion

Recent research has increasingly focused on the bidirectional communication between the gut and the brain in neurological disorders, particularly epilepsy. While investigations of GM in DEE remain in the early stages, especially regarding IESS and DS, emerging evidence suggests a potential link between GM alterations and IESS as well as its role in the response to ACTH. Moreover, preclinical studies have shown that certain non‐convulsant interventions, such as antibiotics, the KD, and PRO, can modulate GM communities and mitigate spasms. Although incorporating PRE into the KD did not enhance seizure control compared to the KD alone, it did ameliorate GM community. In the DS model, mice exhibited dysbiosis and distinct alterations in GM composition. However, despite these changes, the KD failed to improve seizure control in DS mice.

Overall, further studies integrating multi‐omics approaches that combine metagenomics, metabolomics, and immunophenotyping analysis in large longitudinal pediatric populations may pave the way for novel therapeutic strategies aimed at restoring GM balance and improving clinical outcomes in DEEs.

## Author Contributions

**Takwa Ammar:** conceptualization, data curation, investigation, methodology, writing – original draft. **Fatma Abdelhedi:** conceptualization, investigation, methodology, writing – review and editing. **Leila Ammar keskes:** supervision, validation, writing – review and editing. **Chahnez Charfi Triki:** supervision, validation, writing – review and editing.

## Conflicts of Interest

The authors declare no conflicts of interest.

## Peer Review

The peer review history for this article is available at https://www.webofscience.com/api/gateway/wos/peer‐review/10.1111/ejn.70234.
